# Body Composition in Patients with Follicular Lymphoma: Asso-Ciations between Changes in Radiomic Parameters in Patients Treated with R-CHOP-like and R-B Regimens: LyRa 01F

**DOI:** 10.3390/cancers15040999

**Published:** 2023-02-04

**Authors:** Fabiana Esposito, Maria Rosaria Pascale, Cristiano Tesei, Paola Elda Gigliotti, Alessandra Luciano, Cecilia Angeloni, Massimiliano Marinoni, Federico Meconi, Roberto Secchi, Alberto Patanè, Massimiliano Postorino, Maria Cantonetti, Guglielmo Manenti

**Affiliations:** 1 Hematology, Department of Biomedicine and Prevention, University Tor Vergata, 00133 Rome, Italy; 2 Department of Diagnostic Imaging and Interventional Radiology, University of Rome Tor Vergata, 00133 Rome, Italy; 3 Hematolofy, Fondazione Policlinico Tor Vergata, 00133 Rome, Italy; 4 Department of Biomedicine and Prevention, University of Rome Tor Vergata, 00133 Rome, Italy

**Keywords:** follicular lymphoma, R-CHOP, R-B, PET/CT, body composition, bone mineral density

## Abstract

**Simple Summary:**

In this study, we assessed changes in bone mineral density at disease onset and at the end of therapy in follicular lymphoma patients treated with R-CHOP and R-Bendamustine regimens. Another aim was to assess whether low bone mineral density at the onset of the disease is an unfavorable prognostic index. We also evaluated changes in other radiomic parameters, such as the musculoskeletal index, visceral adipose tissue, and subcutaneous adipose tissue in patients treated with R-Bendamustine and R-CHOP, with the latter containing high doses of steroids, which we know are associated with negative bone balance, a loss of muscle mass, and metabolic and cardiovascular issues. This is particularly relevant in elderly patients, in whom these changes could have an important impact on outcomes and the quality of life. As R-CHOP and R-Bendamustine are two effective treatment regimens in follicular lymphoma, this study, together with other clinical-laboratory parameters, could help in the choice of therapy.

**Abstract:**

In patients with follicular lymphoma (FL), therapeutic advances have led to improved survival, and within this framework, it is important to identify treatment strategies offering a better quality of life. Using (18)F-fluorodeoxyglucose positron emission tomography/computed tomography (PET/CT), in patients treated with R-CHOP-like or R-Bendamustine regimens, we assessed changes in the bone mineral density (BMD), musculoskeletal index (SMI), visceral adipose tissue (VAT), and subcutaneous adipose tissue (SAT) at disease onset and at the end of therapy. We evaluated whether the high-steroid regimen could lead to more significant radiological changes than those induced by the steroid-free regimen and whether a low BMD at disease onset is an unfavorable prognostic index. Seventy-nine patients between 60 and 80 years old with a new diagnosis of FL were included in the study. Evaluation of Delta values (pre- and post-therapy mean values) in the two immunochemotherapy regimens showed differences in radiomic parameters within the two patient cohorts. The R-CHOP-like regimen was associated with a significant reduction in BMD, an increase in SAT and VAT, and a reduction in skeletal muscle density (SMD) and SMI. Moreover, patients with high FLIPI showed a BMD below the cut-off value. This study represents the first study demonstrating a prognostic correlation between FLIPI and low BMD.

## 1. Introduction

Follicular Lymphoma (FL) is the most common indolent lymphoproliferative disorder [1] and the second most frequent histological subtype among Non-Hodgkin’s Lymphomas (NHLs) after Large B-cell Lymphoma (DLBCL) in Western Europe [2,3].

This subtype is phenotypically, genetically, and clinically heterogeneous, and its course varies from an indolent type of lymphoma, requiring only “watch and wait”, to a more aggressive one, requiring first-line systemic immunochemotherapy treatment [3].

The 2016 WHO classification uses a 1–3 grading system based on the increase in the number of centroblasts counted per high-power field (hpf). FL grade I has 0 to 5 centroblasts per hpf, FL grade 2 has 6 to 15 centroblasts/hpf, and FL grade 3 has more than 15 centroblasts/hpf. Grade 3 has been divided into grade 3A, in which centrocytes are present, and grade 3B, which has only centroblasts. Grade 3B is clinically and biologically more similar to diffuse large B-cell lymphoma and is treated as such [2,4]. Recently, the fifth edition of the WHO introduced a significant revision of FL, making a distinction between the classical form (cFL) and follicular large B-cell lymphoma (FLBL), with a close and biological relationship with DLBCL and FL with uncommon features (uFL), which comprises two subsets: One with a cytological variant ’blastoid’ or ’large centrocyte’ and the other with a predominantly diffuse variant [5].

For prognostic purposes, the Follicular Lymphoma International Prognostic Index (FLIPI) is the most widely used score in FL and uses simple clinical parameters, such as age, Ann Arbor stage, hemoglobin value, Lactic Dehydrogenase (LDH), and the number of lymph nodes involved [6].

In patients requiring treatment, two other scores such as FLIPI2 and FIRST FLIPI have been suggested. More recently, a genetic risk score (m7FLIPI) has been added in combination with the FLIPI score, which considers seven candidate genes [7]. However, none of these indices guide the choice of therapy in common clinical practice.

In patients with advanced FL who require treatment and in whom complete remission (CR) and long progression-free survival (PFS) are sought, rituximab in combination with anthracycline-based chemotherapy treatments (R-CHOP-like) such as cyclophosphamide doxorubicin, vincristine, and prednisone (CHOP), or R-COMP (rituximab-cyclophosphamide-liposomal doxorubicin-vincristine-prednisone), or the combination of rituximab with bendamustine(R-B), should be used as first-line treatments [8,9,10].

In patients with grade 3B follicular lymphomas, or if there are clinical signs of transformation, it is preferable to use immunochemotherapy regimens containing anthracyclines (R-CHOP-like) [3].

Several studies have compared the two regimens, and no superiority in terms of therapeutic effect has been found between R-CHOP and R-B [10], and thus, often, in clinical practice, the choice of treatment is based on clinical parameters at the onset of the disease, such as the patient’s age and fitness, the presence of cardiological comorbidities (cardiotoxic effect of anthracyclines), or the refusal of treatment that may cause alopecia

18FDG PET/CT is the current standard for staging and assessing therapeutic response in FL [11].

The images obtained from CT scans performed during PET/CT can be used to assess BMD by placing a region of interest (ROI) on the vertebral body of the first lumbar vertebra (L1) and referring to standardized values to define osteopenia and osteoporosis, based on gender and age [12]. In a CT scan, skeletal muscle density (SMD), as a measure of attenuation, represents the degree of lipid deposition in skeletal muscle and can be a surrogate for muscle quality. Furthermore, advanced software allows the extraction of quantitative measures of the skeletal muscle area (SMA), subcutaneous adipose tissue (SAT), and visceral adipose tissue (VAT), even retrospectively [13,14]. From these values and when knowing the patient’s height, the skeletal muscle index (SMI) can be calculated as SMA divided by the square of their height. Due to the significant variation in body composition between males and females, sex (gender)-specific cut-offs of the skeletal muscle index for non-obese men and women have been proposed, based on the body mass index (BMI) category and gender [14].

Currently, most of the radiomic studies that have investigated PET/CT as a tool for personalizing treatment and as a predictor of progression-free survival (PFS) and overall survival (OS) have been developed primarily for DLBCL [15,16,17,18,19].

Several studies have shown an increased loss of bone mass and increased risk of fractures in patients treated for NHL with high doses of corticosteroids [20,21,22,23,24].

The main endpoint of this experimental study is to evaluate changes in the body composition in FL patients in PET/CT at the end of the two different immunochemotherapies because, in the future, these parameters could help us in the therapy choice and assess a possible prognostic value of radiomic parameters at disease onset.

## 2. Materials and Methods

### 2.1. Study Population

The study population was retrospectively selected from a database of patients with FL, referred to our Institute between January 2011 and May 2019, with a post-treatment follow-up of at least 30 months. The sample included 79 patients with newly diagnosed FL grades 1–3A according to the 2016 WHO classification, treated with first-line chemotherapy regimens R-CHOP-like or R-B. Patients who relapsed within 30 months were also included in the analysis.

Patient inclusion criteria included an age range of 60–80 years, a baseline 18FDG PET/CT scan suitable for radiomic analysis performed at the Department of Diagnostic Imaging Radiology of the Policlinico Tor Vergata, first-line treatment with six cycles R-CHOP: Rituximab 375 mg/m^2^, Cyclophosphamide 750 mg/m^2^, Doxorubicin 50 mg/m^2^ (liposomal Doxorubicin 50 mg/m^2^ in R-COMP), Vincristine 1.4 mg/m^2^(for a maximum of 2 mg total dose) on day 1 and Prednisone 100 mg for 5 days every 21 days) or six cycles of R-B: Rituximab 375 mg/m^2^ on day 1 and Bendamustine 90 mg/m^2^ on days 1 and 2 of the cycle every 28 days.

In order to ensure the population was homogeneous, patients diagnosed with FL grade 3B, patients who had not undergone baseline or reassessment 18FDG PET/CT after immunochemotherapy at our Institute, and patients for whom it was impossible to access the images or examine the radiomic parameters considered were excluded. Table 1 summarizes the characteristics of the evaluated patients.

The database included 150 patients with FL in the 60–80 age range, and 71 were excluded because they did not fulfil the inclusion criteria. Early-stage patients who had performed only locoregional radiotherapy or for whom treatment criteria were not matched were excluded from our study. Patients who had performed immunochemotherapy treatments other than the R-CHOP or R-B regimen were also excluded.

For each patient, PET/CT was performed at the beginning of immunochemotherapy treatment and one month after its completion. For each patient, we considered descriptive parameters such as age and sex and clinical criteria such as the date of diagnosis, histology, Ann Arbor stage, performance status according to the ECOG scale, weight, and height; from these we calculated FLIPI, response to the end of therapy, the date of progression (if present), and the date of last contact or death. Finally, from the radiomic parameters we calculated and measured BMD, SMI, VAT, SAT, SMD, and SMA at onset and after chemoimmunotherapy treatment.

### 2.2. Radiomics Parameters 

Quantitative measurements of BMD were taken from CT images at the level of the 1st lumbar vertebra. The body composition profile was performed at the level of the 3rd lumbar vertebra, including SMD, SMA, SMI, SAT, and VAT on both pre-treatment baseline CT scans and end-of-treatment CT scans. A radiologist extrapolated some parameters from the low-dose CT co-registration PET scan, using GE Adw Cross X software on a dedicated workstation (ADVANTAGE WORKSTATION 4.4. GE MEDICAL SYSTEMS).

**Figure 1:**
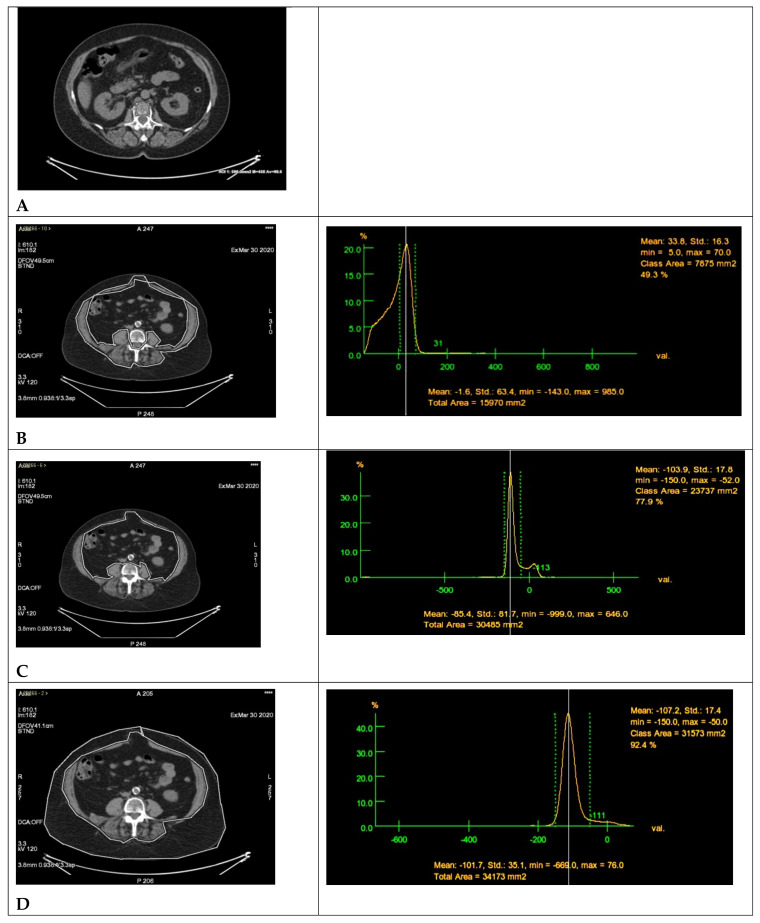
(**A**) BMD L1: BMD was measured in Hounsfield Units (HUs) and calculated with the Low-dose CT co-registration PET scan slice passing through the middle III of the L1 soma, placing an ROI with an area of approximately 600 mm^2^. (**B**) SMA L3: Low-dose CT co-registration PET scan with the slice passing through the middle III of L3 soma (on the left) and the ROI tissue density percent trend graphic (on the right) extrapolated using GE Adw Cross X software on a dedicated workstation (ADVANTAGE WORKSTATION 4.4. GE MEDICAL SYSTEMS). The SMA (measured in mm^2^, then converted to cm^2^) was calculated by drawing a freehand ROI at the slice through the middle III of the L3 soma including all abdominal, lumbar, and paravertebral muscles and selecting, within the aforementioned ROI, only those tissues that had muscle density. In the case shown, the SMA is 7875 mm^2^, represented in the graphic as the area of tissue with density range between the two green dotted lines (maximum density of 70 HU, minimum density of 5 HU), with mean density of 33.8 HU (white line). (**C**) VAT L3: Low-dose CT co-registration PET scan at the slice passing through the middle III of L3 soma (on the left) and the ROI tissue density percent trend graphic (on the right) extrapolated using GE Adw Cross X software on a dedicated workstation (ADVANTAGE WORKSTATION 4.4. GE MEDICAL SYSTEMS). The VATA (measured in mm^2^ then converted to cm^2^) was measured by drawing a free-hand ROI at the slice through the middle III of the L3 soma, which included the abdominal region, excluding the abdominal muscle region and the SAT and selecting, within the aforementioned ROI, only tissues with fat density. In the case shown, the VATA is 23,737 mm^2^, represented in the graphic as the area of tissue with density range between the two green dotted lines (maximum density of −150 HU, minimum density of −52 HU), with mean density of −103.9 HU (white line). (**D**) SAT L3: Low-dose CT co-registration PET scan at the slice passing through the middle III of L3 soma (on the left) and the ROI tissue density percent trend graphic (on the right) extrapolated using GE Adw Cross X software on a dedicated workstation (ADVANTAGE WORKSTATION 4.4. GE MEDICAL SYSTEMS). The SATA (measured in mm^2^, then converted to cm^2^) was measured by drawing a freehand ROI at the slice through the middle III of the L3 soma, which included the abdominal wall tissues external to the abdominal muscle region and selecting, within the aforementioned ROI, those with adipose density. In the case shown, the SATA is 31,573 mm^2^, represented in the graphic as the area of tissue with density range between the two green dotted lines (maximum density of −150 HU, minimum density of −50 HU), with mean density of −107.2 HU (white line).

BMD, measured in Hounsfield Units (HUs), was calculated at the slice passing through the middle third of the L1 soma, using an ROI with an area of 600 mm^2^ (Figure 1A). For the SMD, SMA, VATA (area of visceral adipose tissue), and SATA (area of subcutaneous adipose tissue) calculation, the slice through the middle third of the L3 vertebral soma was used as the reference slice, as proposed by Xiao et al. [25]. 

SMD was assessed in HU, with an ROI within an area of 400 mm^2^ drawn at the psoas muscle. 

SMA was measured by drawing a freehand ROI comprising all abdominal, lumbar, and paravertebral muscles and selecting, within that ROI, only those tissues with muscle density (Figure 1B).

The SMI (skeletal muscle index, cm^2^/m^2^) was calculated as SMA (cm^2^)/patient height (m)^2^. 

The VATA (visceral adipose tissue area, cm^2^) was measured by drawing a freehand ROI including the abdominal region, excluding the abdominal muscle region and SAT (subcutaneous adipose tissue), and selecting, within the aforementioned ROI, only tissues with adipose density (−150 to −50 HU approximately) (Figure 1C).

The SATA (subcutaneous adipose tissue area, cm^2^) was calculated by drawing a freehand ROI that includes the tissues of the abdominal wall outside the abdominal muscular region and selecting, within this ROI, those with adipose density (Figure 1D).

### 2.3. Statistical Analysis

A preliminary analysis to select a proper sample size was performed, referring to published data [23]. Assuming a normal distribution of BMD and a power of 0.90, with alpha = 0.05, the total sample size should have been *n*=78. 

All continuous variables were first evaluated by the Shapiro–Wilk Test. Continuous variables are expressed as the median or mean ± SD; categorical variables are expressed as percentages. Delta (Δ) values (post-therapy mean value–pre-therapy mean value) and delta percentages were performed to evaluate the change in the pre–post-therapy values between radiomic groups. Boxplots were used to show BMD, SAT, and VAT values between pre- and post-therapy.

In the univariate analysis, parametric and non-parametric tests were performed for comparisons between groups (Chi-Squared and Fisher Exact tests in the case of categorical variables; Student’s *t*-test and Mann−Whitney tests in the case of continuous variables and Wilcoxon signed-rank tests in the case of ordinal variables). Receiver operating characteristic curves (ROCs) were implemented to identify the optimal cut-off for BMD, SAT, and VAT variables with the most effective measure of sensitivity and specificity in the FLIPI score (low vs. high) pre-therapy. Youden’s Index was calculated as (sensitivity + specificity) − 100 using the ROC curves. All tests were two-sided, accepting *p* < 0.05 as indicating a statistically significant difference. Confidence intervals were calculated at the 95% level. Progression-Free Survival (PFS) distributions were estimated using the Kaplan–Meier product limit estimator. Differences in PFS curves were evaluated using the Log-Rank test. Cox regression models were performed in univariate analyses to assess the effect of FLIPI scores and the BMD cut-off on progression-free survival (PFS).

All analyses were performed using the R system software (R Foundation for Statistical Computing c/o Institute for Statistics and Mathematics, Wirtschaftsuniversität, 1020 Wien, Austria), GraphPad Prism ver. 9.0.0, G*Power (Release 3.1.9.6) and Microsoft Corporation (2022) Microsoft Excel for Mac, retrieved from https://office.microsoft.com/excel, 11 January 2023.

## 3. Results

The minimum calculated sample size was 39 patients in each group (the minimum total of patients for the study was 76) with probability (1 − β) greater than 0.9 and an effect size δ ≥ 0.75, with a two-way criterion allowing for a maximum type I error rate α = 0.05.

The main demographic and clinical characteristics of the eligible patients are summarized in Table 1. Seventy-nine patients with follicular NHL were treated with immunochemotherapy: 48 according to the R-CHOP scheme and 31 patients according to R-B therapy. 

The two groups were homogeneous in terms of age at treatment (66 years vs. 70 years) and weight, height, and BMI parameters. The male-to-female ratio (M:F) is unbalanced toward women in the R-CHOP group (M/F ratio = 0.7) and toward men in the R-B group (M/F ratio = 1.4).

In both patient cohorts, the FLIPI score showed a higher rate in the intermediate risk category (58.3% in R-CHOP and 54.8% in R-B). Patients with low-risk FLIPI scores occurred at a lower rate (2.1% in R-CHOP and 9.7% in R-B), which confirms the correct indication for the start of chemotherapy treatment.

**Figure 2:**
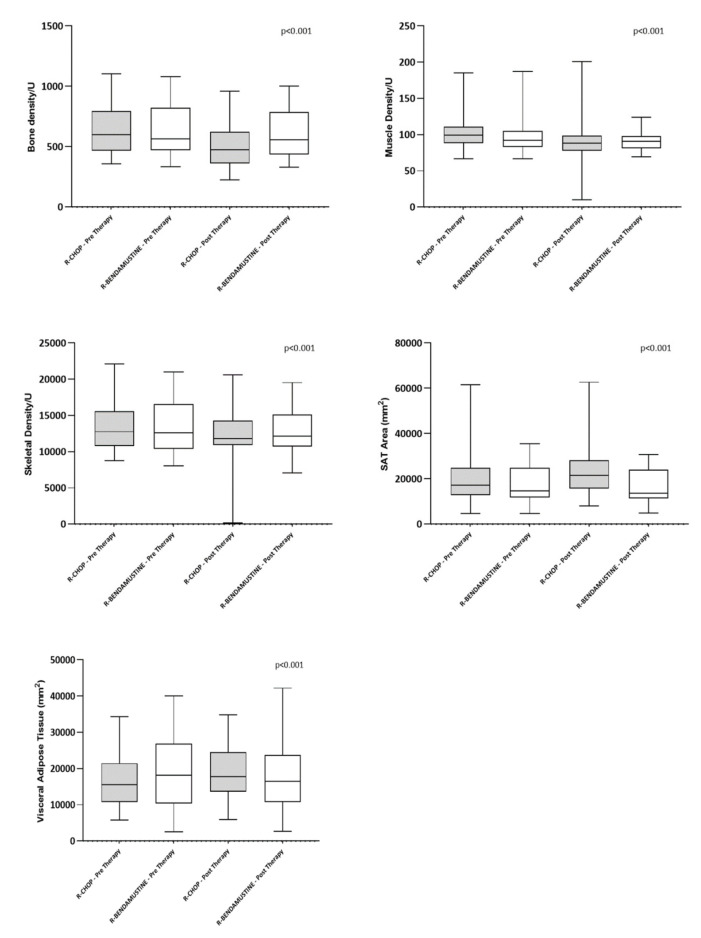
Boxplot of BMD, PMD, SMA, SAT, and VAT at baseline and after chemotherapy.

The pre- and post-therapy radiomic data are summarized in Table 2 and Figure 2. These show that the median pre-therapy values were overlapping for both study groups for the radiomic variables.

**Figure 3:**
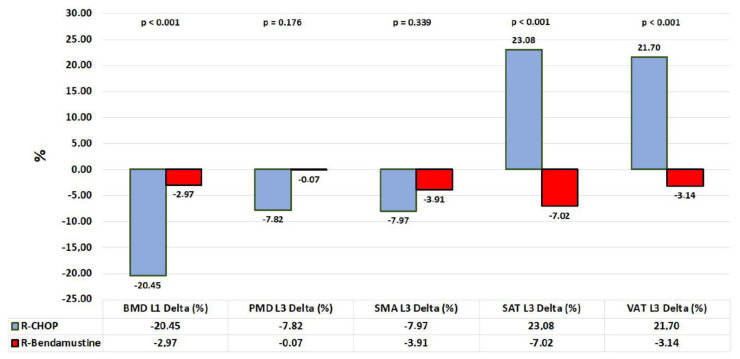
Graphical representation of “delta” mean values stratified by R-CHOP vs. R-Bendamustine therapy.

Furthermore, the difference in mean values pre–post-therapy of BMD, SMD, and SMA in the R-CHOP patient group is lower than in the R-B group (Δ(%) = BMD/R-CHOP = −20.45%, Δ(%) = BMD/R-B = −2.97%, *p* < 0. 001; Δ(%) = SMD/R-CHOP = −7.82, Δ(%) = SMD/R-B = −0.07%, *p* = 0.176; Δ(%) = SMA/R-CHOP = −7.97%, Δ(%) = SMA/R-B = −3.91%, *p* = 0.339). In addition, there is an increase in SAT and VAT values in the R-CHOP group; for VAT: Δ(%) = VAT/R-CHOP: 21.70%, Δ(%) = VAT/R-B: −3.14%, *p* < 0.001; for SAT: Δ(%) = SAT/R-CHOP: 23.08%, Δ(%) = VAT/R-B: −7.02%, *p* < 0.001; see Table 3 and Figure 3. 

**Figure 4:**
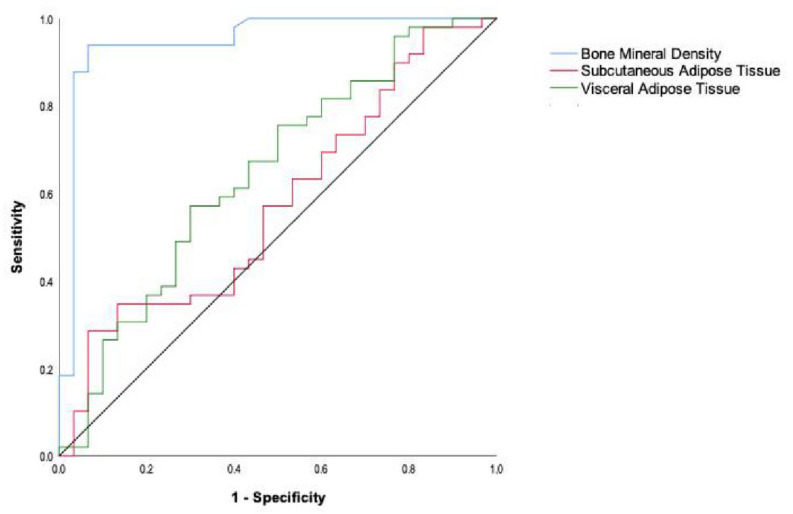
Prediction analysis of radiomic parameters and the outcomes of patients with low or high FLIPI Scores.

ROC curves were used to analyze the radiomic parameters (BMD, SAT, and VAT) significantly different between low FLIPI Scores (FLIPI Score 1 and 2) and high FLIPI Scores (FLIPI Score ≥ 3). The ROC curves of certain single parameters are shown in Figure 4. The minimum sum of squares of the false-positive rate and the false-negative rate was used as a reference for the choice of the optimal cut-off value. The results have shown that BMD was the best single parameter for predicting the prognosis of patients, with an area under the ROC curve (AUC) of 0.948 [95% confidence interval (CI) 0.889–0.996]. When the BMD cut-off value was 522, the sensitivity and specificity of BMD for predicting the FLIPI Score as low or high were 93.9% and 93.3%, respectively (Table 4).

In addition, we have observed that percentages of patients who have high FLIPI Scores are below the cut-off value of BMD for R-CHOP and B-R (94.7% and 90.9%, *p* < 0.001), while for patients who have low FLIPI Scores, the cut-off values of BMD for R-CHOP and R-B are higher (96.5% and 85.0%, *p* < 0.001) (Table 5).

**Figure 5:**
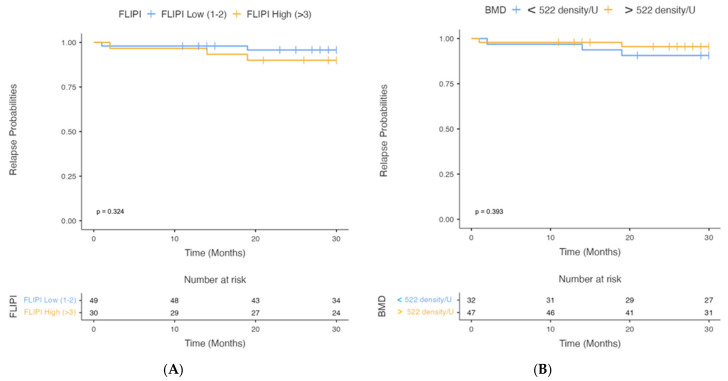
Analysis of PFS according to FLIPI Score (**A**) and BMD Cut-off (**B**).

Analysis of PFS according to FLIPI Score (low vs. high) showed a higher level of high FLIPI Scores (*p* = 0.324), which never reached the median (after 30 months of observation, 95.9% of patients with a low FLIPI Score and 90.0% of patients with a high FLIPI Score did not relapse) (Figure 5A).

Analysis of PFS according to the cut-off of BMD (<522 density/U vs. >522 density/U) showed a higher level of BMD < 522 density/U (*p* = 0.393), which never reached the median (after 30 months of observation, 90.6% of patients with BMD < 522 density/U and 95.7% of patients with BMD > 522 density/U did not relapse) (Figure 5B).

## 4. Discussion

In most cases, FL is still an incurable disease, and [3] despite the good response to new therapies with a significant improvement in prognosis, relapses are frequent in its course and histological transformation to DLBCL is also possible [26].

In recent decades, therapeutic advances offer FL patients a perspective of prolonged survival, therefore it is important to identify treatment strategies that offer a better quality of life. 

Recently, interest has grown in seeking functional parameters derived from staging PET/CT, and several studies have suggested, with increasing evidence, the potential prognostic value of quantitative parameters when staging PET/CT in patients with different types of non-Hodgkin’s lymphoma [27,28,29,30].

To date, for patients with FL in need of treatment, there are two R-chemotherapy approaches, R-CHOP and R-B, both effective and equally indicated as first-line therapy in patients requiring systemic treatment [9,10]. In addition, the choice to perform one of the two regimens is often based on factors concerning the patient’s age and fitness, as well as the presence of cardiological comorbidities (cardiotoxic effect of anthracyclines [31], the presence of a caregiver, and the refusal of hair loss treatment [10]. 

Our study arises from the hypothesis that the identification and application of radiomic patterns can improve our ability to make therapeutic choices. 

The studies that have investigated this issue have focused on other disciplines such as oncology and rheumatology. In the field of hematology, there are few data in the literature on the effect of chemotherapy on bone metabolism in adult patients with NHL. The available data are those relevant to patients diagnosed with DLBCL and treated according to the R-CHOP regimen [21,22,23,24]. In a prospective study, Anargyrou et al. suggest that first-line chemotherapy leads to high bone turnover, increased bone loss, and reduced BMD at the level of the lumbar and femoral [24]. Likewise, Cabanillas et al., in a large retrospective study of patients over 65 years of age with NHL, showed a significant increase in fractures and osteoporosis [21]. 

Furthermore, a significant loss of bone mineral density after R-CHOP(-like) treatment was observed in 111 patients with DLBCL. Glucocorticoid-induced osteoporosis may therefore have an impact on survival for a large fraction of patients with DLBCL with durable remissions [23].

Our study is the first to compare radiomic parameters in two cohorts of patients with FL treated with two different immunochemotherapy regimens. 

To date, there are no studies on this subject, perhaps also due to the difficulty in collecting data in a heterogeneous population. Therefore, to reduce potential selection bias, we chose to collect patients with a defined age range (60–80 yr), classified by grading and risk class according to FLIPI.

Our results show a marked reduction in BMD in the group of patients who received R-CHOP compared to the ones who received R-B.

This could be due to the administration of the first regimen of corticosteroids, which are known to be associated with an increased risk of reduced BMD, and thus osteoporosis [32,33].

The negative effect that glucocorticoids have on bone mass, and the subsequent risk of fractures, has been documented primarily in patients with Rheumatoid Arthritis in whom average daily dosages generally do not exceed 5–7.5 mg prednisone [34,35].

However, while the effects in patients who receive corticosteroids continuously and for long periods have been studied, only in recent years has the focus been moving to the patient population receiving intermittent doses of steroid-based chemotherapies for a limited number of days, as is the case in the treatment of lymphoid disorders.

Most LNH regimens, such as CHOP and COMP, are administered every 3 weeks for 6 cycles, include prednisone 100 mg orally once a day for 5 days, and are associated with a high incidence of vertebral fractures [21,22]. The pathogenesis of glucocorticoid-induced osteoporosis is multifactorial, with mechanisms partly involving bone cells, and mediated by local and systemic interactions between hormones, growth factors, and cytokines. With regard to the latter, the RANKL/OPG (receptor activator of nuclear factor kappa B-ligand/osteoprotegerin) system is thought to be primarily responsible for the reduction in bone mass and the rapid increase in fracture risk [36,37,38,39].

Indeed, glucocorticoids induce osteoblast expression of RANKL and inhibit osteoblastic production of osteoprotegerin resulting in an increase in osteoclastic activity. Further osteoclast-stimulating action is significantly linked to certain cytokines with pro-inflammatory activity, such as TNF-α and Interleukins 1, 6, and 17 (IL-1, IL-6, IL-17) [40,41,42,43].

According to some studies, cytokines also play an important role in the pathogenesis of lymphoma and are partly responsible for systemic symptoms [44,45]. 

Elevated serum or tissue cytokine levels may contribute to clinical and histopathological alterations in NHL. Moreover, it has been shown that levels of serum TNF-α, IL-2, and serum CD44 (sCD44) are all increased in NHL and correlate with tumor burden, the presence of symptoms, and other clinical and laboratory variables [46,47].

Our study showed a correlation between initial BMD and FLIPI: Patients who had a high FLIPI (3–4) had an initial BMD lower than 522 HU, which could refer to a higher cytokine release in aggressive diseases [48,49,50]. 

PFS analysis according to FLIPI and BMD, although only assessed at 30 months and not reaching the median, showed a lower PFS in patients with high FLIPI and low BMD. After 30 months of observation, 95.9% of patients with a low FLIPI Score and 90.0% of patients with a high FLIPI Score did not relapse.

PFS analysis based on the BMD cut-off has shown, after 30 months of observation, that 90.6% of patients with BMD < 522 density/U and 95.7% of patients with BMD > 522 density/U had no relapse.

In addition, the R-CHOP chemotherapy includes the use of drugs such as vincristine, which can cause polyneuropathy as a side effect, with decreased physical activity and increased risk of falls [51].

Neuropathy affects the perception of sensory signals and proprioception, causing gait instability. If we consider the altered muscle mass, with the resulting sarcopenia, and the use of corticosteroids, these patients inevitably have an increased risk of accidental falls [52].

Corticosteroids are also well known to induce most of the main features of metabolic syndromes such as hyperglycemia, insulin resistance, dyslipidemia, hepatic steatosis, and obesity, having a significant effect on the quality of life of patients [53].

On this topic, some studies have also been undertaken with hematological patients, as demonstrated in surviving children with acute lymphoblastic leukemia [54]. The effects of steroid therapy translate into an increased appetite, water retention, and fat redistribution in certain body districts such as the abdomen, neck, and face, resulting in the typical ’full moon’ shape. 

The results of our study confirm these observations, as when evaluating the SAT L3 before and after therapy in the two patient cohorts as secondary objectives, we observed a high delta value in the R-CHOP group and a low delta in post-therapy SAT in the R-B group. 

We have hypothesized that the reduction in the R-B group could have been related both to the neoplastic disease itself, due to the increase in metabolic cell-induced spending by the tumor cells with a persistence of the chronic inflammatory state, and to the effect of chemotherapy, due to the reduced food intake, decreased appetite, and altered digestive capacity. This process would not be offset by the lipogenic action of cortisone, which is used in the R-CHOP group and is responsible for the increase in SAT L3.

With regard to VAT L3, the results of our study show a post-therapy trend similar to SAT L3. One reason could be attributed to cytokine release by lymphoma cells, which is responsible for systemic symptoms, including weight loss [44,45]. 

In the R-B group, this effect would not be balanced by the anti-inflammatory action of glucocorticoids, which down-regulate pro-inflammatory cytokines such as IL-1α, IL-1β, IL-2, IL-3, IL-5, IL-6, IL-8, IL-12, IFN-γ, TNF-α, and GMCSF [53,55]. This would lead, over time, to a reduction in visceral fat.

Furthermore, preliminary studies have shown that high blood glucose levels (which occur after taking corticosteroids) would attenuate the increase in IL-6 levels in plasma [56,57].

## 5. Conclusions

Our study provides initial proof of the hypothesis of a significant change in the body composition of patients treated with R-CHOP compared to those treated with R-B. For the time being, we have provided important descriptive aspects that we can consider in the future choice of therapy between two effective immunochemotherapies, especially in frail patients.

However, the study was conducted as a retrospective analysis, with the limitation of being monocentric and addressing a population observed for a 30-month follow-up period, which did not allow us to assess OS. PFS, although evaluated for a very short time period (30 months), showed higher value in patients with low FLIPI and high BMD. During our analysis, we observed a correlation between BMD and FLIPI. Indeed, the correlation was very significant. This, in our opinion, could have been influenced by the greater cell turnover and cytokine release observed in the more advanced stages of the disease. This could also be influenced by unknown factors. Further analysis is needed to consolidate our initial observations.

Therefore, we will need prospective studies in the future, extended to a larger population, with a follow-up longer than 30 months, which must include questionnaires on patients’ quality of life to assess whether these radiomic parameters can really provide an additional reference tool for the choice of therapy in clinical practice.

## Figures and Tables

**Table 1:** Presentation characteristics stratified by R-CHOP vs. R-Bendamustine therapy.

Population (*n* = 79)	R-CHOP (*n* = 48)	R-Bendamustine (*n* = 31)	*p*-Value
**Median age, years (range)**	66 (52–77)	70 (59–81)	0.058
**Male:Female ratio**	0.7 (20:28)	1.4 (18:13)	0.154
**Median height, cm** **± SD (range)**	166.3 ± 9.1 (150.0–189.0)	167.5 ± 7.9 (150.0–184.0)	0.556
Median height, cm ± SD (range)-Male	170.5 ± 7.5 (162.0–189.0)	172.0 ± 5.7 (160.0–184.0)	0.652
Median height, cm ± SD (range)-Female	162.0 ± 6.8 (150.0–173.0)	160.0 ± 6.4 (150.0–172.0)	0.988
**Median weight, Kg** **± SD (range)**	75.8 ± 21.2 (48.0–187.0)	72.0 ± 14.2 (48.0–97.0)	0.380
Median weight, Kg ± SD (range)-Male	79.5 ± 13.0 (65.0–121.0)	77.5 ± 12.5 (60.0–97.0)	0.810
Median weight, Kg ± SD (range)-Female	66.5 ± 25.3 (48.0–187.0)	63.0 ± 10.4 (48.0–80.0)	0.133
**Median BMI** **± SD (range)**	22.8 ± 6.3 (16.0–56.7)	21.4 ± 3.8 (14.8–28.8)	0.510
Median BMI ± SD (range)-Male	22.6 ± 3.4 (19.7–34.0)	22.7 ± 3.7 (17.1–28.8)	0.915
Median BMI ± SD (range)-Female	20.0 ± 7.8 (16.0–56.7)	19.9 ± 2.8 (14.8–24.4)	0.161
**Grading**			
G1	4.2% (2/47)	16.1% (5/31)	0.241
G1-G2	14.9% (7/47)	16.1% (5/31)	
G2	49.0% (23/47)	42.0% (13/31)	
G2-G3A	6.4% (3/47)	12.9% (4/31)	
G3A	25.5% (12/47)	12.9% (4/31)	
**FLIPI Score**			
Low	2.1% (1/48)	9.7% (3/31)	0.322
Intermediate	58.3% (28/48)	54.8% (17/31)	
High	39.6% (19/48)	35.5% (11/31)	

SD: Standard deviation; Mann–Whitney test, Student’s *t*-test, Wilcoxon signed-rank tests, Chi-square test. *p* < 0.05 significant.

**Table 2:** Pre–post-therapy radiomic values.

Population (*n* = 79)	R-CHOP			R-Bendamustine		
Primary Endpoint	Pre-Therapy	Post-Therapy	*p*-Value	Pre-Therapy	Post-Therapy	*p*-Value
**Mean ± SD Bone Mineral Density L1 (density/U)**	632.79 ± 194.99	506.94 ± 180.25	<0.001	632.58 ± 205.80	608.39 ± 193.82	0.060
**Mean ± SD Skeletal Muscle Density L3 (density/U)**	101.38 ± 22.57	91.19 ± 25.13	0.017	95.55 ± 22.20	92.03 ± 14.35	0.432
**Mean ± SD Skeletal Muscle Area L3 (mm^2^)**	13,283.23 ± 3015.18	12,200.69 ± 3622.12	0.005	13,501.45 ± 3387.46	12,842.10 ± 3001.80	0.042
**Mean ± SD Subcutaneous Adipose Tissue L3 (mm^2^)**	19,404.23 ± 10,027.24	22,731.52 ± 9839.26	<0.001	17,935.26 ± 7897.96	16,569.84 ± 7601.68	0.006
**Mean ± SD Visceral Adipose Tissue L3 (mm^2^)**	16,445.08 ± 7067.21	19,388.00 ± 7229.37	<0.001	18,591.36 ± 9731.36	17,557.81 ± 9150.47	0.053

SD: Standard deviation; Mann–Whitney test, Student’s *t*-test. *p* < 0.05 significant.

**Table 3:** Descriptive characteristics stratified by R-CHOP vs. R-Bendamustine therapy: Radiomics data-delta.

Population (*n* = 79)	R-CHOP (*n* = 48)						R-Bendamustine (*n* = 31)						
	Mean ± SD			95% CI			Mean ± SD			95% CI			*p*-Value
**BMD L1 Delta**	−125.85	±	72.64	(−146.40	–	−105.30)	−24.19	±	68.84	(−48.42	–	0.04)	<0.001
**BMD L1 Delta (%)**	−20.45	±	11.05	(−23.58	–	−17.32)	−2.97	±	11.51	(−7.02	–	1.08)	<0.001
**SMD L3 Delta**	−10.19	±	28.48	(−18.25	–	−2.13)	−3.52	±	24.56	(−12.17	–	5.13)	0.232
**SMD L3 Delta (%)**	−7.82	±	24.96	(−14.88	–	−0.76)	−0.07	±	24.14	(−8.57	–	8.43)	0.176
**SMA L3 Delta (mm^2^)**	−1082.54	±	2541.00	(−1801.38	–	−363.70)	−659.36	±	1724.97	(−1266.58	–	−52.14)	0.641
**SMA L3 Delta (%)**	−7.97	±	21.70	(−14.11	–	−1.83)	−3.91	±	11.15	(−7.84	–	0.02)	0.339
**SAT L3 Delta (mm^2^)**	3327.29	±	2792.39	(2537.33	–	4117.25)	−1365.42	±	2596.55	(−2279.46	–	−451.38)	<0.001
**SAT L3 Delta (%)**	23.08	±	24.71	(16.09	–	30.07)	−7.02	±	16.28	(−12.75	–	−1.29)	<0.001
**VAT L3 Delta (mm^2^)**	2942.92	±	2692.75	(2181.15	–	3704.69)	−1033.55	±	2851.65	(−2037.39	–	−29.71)	<0.001
**VAT L3 Delta (%)**	21.70	±	21.36	(15.66	–	27.74)	−3.14	±	11.20	(−7.08	–	0.80)	<0.001

Abbreviations. SD: Standard deviation; BMD: Bone Mineral Density; SMD: Skeletal Muscle Density; SMA: Skeletal Muscle Area; SAT: Subcutaneous Adipose Tissue; VAT: Visceral Adipose Tissue. SD: Standard deviation; Mann–Whitney test, Student’s *t*-test. *p* < 0.05 significant.

**Table 4:** Prediction analysis of radiomic parameters of patients with low or high FLIPI Scores.

Variable	AUC	95% CI	Cut-Off Point	Sensitivity (%)	Specificity (%)	Youden’s Index	*p* Value
**BMD**	0.948	0.889–0.996	522	93.9	93.3	0.872	<0.001
**SAT**	0.577	0.446–0.707	25,976	93.3	87.6	0.219	0.537
**VAT**	0.646	0.518–0.774	16,693	70.0	75.7	0.646	0.042

AUC, area under the ROC curve; 95% CI, 95% confidence interval.

**Table 5:** Comparison of FLIPI Score and BMD cut-off results of R-CHOP-like vs. R-B therapy.

Population (*n* = 79)	R-CHOP			R-Bendamustine		
Primary Endpoint	BMD < 522	BMD > 522	*p*-Value	BMD < 522	BMD > 522	*p*-Value
**FLIPI Score Low (1–2)**	3.5% (1/29)	96.5% (28/29)	<0.001	15.0% (3/20)	85% (17/20)	<0.001
**FLIPI Score High (≥3)**	94.7% (18/19)	5.3% (1/19)		90.9% (10/11)	9.1% (1/11)	

**BMD: Bone Mineral Density.**

## Data Availability

The data presented in this study are available on request from the corresponding author.
